# Coverage determinants of breast cancer screening in Flanders: an evaluation of the past decade

**DOI:** 10.1186/s12939-020-01323-z

**Published:** 2020-11-27

**Authors:** L. Ding, S. Jidkova, M. J. W. Greuter, K. Van Herck, M. Goossens, P. Martens, G. H. de Bock, G. Van Hal

**Affiliations:** 1 Department of Epidemiology, University Medical Center Groningen, University of Groningen, Groningen, The Netherlands; 2 Department of Social Epidemiology and Health Policy, University of Antwerp, Antwerp, Belgium; 3 Department of Public Health and Primary Care, Ghent University, Ghent, Belgium; 4 Center for Cancer Detection, Flanders, Belgium; 5 Department of Radiology, University Medical Center Groningen, University of Groningen, Groningen, The Netherlands; 6 Department of Robotics and Mechatronics, University of Twente, Enschede, The Netherlands

**Keywords:** Breast neoplasms, Mammography, Mass screening, Coverage rate, Social determinants of health

## Abstract

**Background:**

Breast cancer (BC) is the most common cancer in women in the developed world. In order to find developing cancers in an early stage, BC screening is commonly used. In Flanders, screening is performed in and outside an organized breast cancer screening program (BCSP). However, the determinants of BC screening coverage for both screening strategies are yet unknown.

**Objective:**

To assess the determinants of BC screening coverage in Flanders.

**Methods:**

Reimbursement data were used to attribute a screening status to each woman in the target population for the years 2008–2016. Yearly coverage data were categorized as screening inside or outside BCSP or no screening. Data were clustered by municipality level. A generalized linear equation model was used to assess the determinants of screening type.

**Results:**

Over all years and municipalities, the median screening coverage rate inside and outside BCSP was 48.40% (IQR: 41.50–54.40%) and 14.10% (IQR: 9.80–19.80%) respectively. A higher coverage rate outside BSCP was statistically significantly (*P* < 0.001) associated with more crowded households (OR: 3.797, 95% CI: 3.199–4.508), younger age, higher population densities (OR: 2.528, 95% CI: 2.455–2.606), a lower proportion of unemployed job seekers (OR: 0.641, 95% CI: 0.624–0.658) and lower use of dental care (OR: 0.969, 95% CI: 0.967–0.972).

**Conclusion:**

Coverage rate of BC screening is not optimal in Flanders. Women with low SES that are characterized by younger age, living in a high population density area, living in crowded households, or having low dental care are less likely to be screened for BC in Flanders. If screened, they are more likely to be screened outside the BCSP.

## Background

Belgium is among the countries with the highest female breast cancer (BC) incidence and mortality worldwide [1]. In 2018, the age-standardized BC incidence and mortality rate of Belgium women were 113.2/100,000 and 22.2/100,000 person-years, respectively, which is higher than the estimate for the whole Western European region (92.6 and 15.5/100,000 person-years, respectively) [1, 2]. While sufficient evidence has indicated that mammography screening has the potential to initiate early diagnosis and treatment for BC and lower BC mortality, the effect of mammography screening relies on the degree to which women participate in screening [3].

In most of the high income countries, women are recommended to participate in an organized BC screening program (BCSP), where quality is warranted by systematic quality control measures [4]. Outside this program, with the aim to screen more of the eligible women, spontaneous screening is also endorsed in some countries, such as in Belgium [5], France [6] and Switzerland [7]. The coverage of BC screening, defined as the percentage of screened women in the total eligible population within the specific interval of routine screening [8], is an important indicator for the evaluation of the effectiveness of screening [4, 9]. However, the average coverage rate in 2016 across OECD countries was only 57.4% [10]. As for Flanders, the coverage rate of BCSP in 2017 was only 49 and 13% was screened outside the BCSP [11].

Many factors have previously been shown to be associated with a reduced coverage level of BCSP. A systematic review summarized that the barriers to BC screening fell into three categories: 1) health care system level barriers, such as lack of health care providers and economic barriers; 2) social barriers, such as lack of social support and cultural norms opposed to BC screening;, and 3) individual level barriers, such as lack of cancer knowledge and beliefs, negative expectations of screening, and distrust of the medical system [12]. However in this review, the majority of the included studies relied on self-reported data, studies with random and convenience samples were pooled, and evidence was only qualitatively synthesized. Many other studies have also provided quantitative evidence on these hampering factors. Among them, economic related barriers were the most commonly studied factors and results showed that low income [13], crowded housing condition [14], unemployment [15] and residing in social-economically deprived areas [16] are predictors of a lower BC screening coverage rate. Lack of a regular health care provider is associated with a reduced coverage rate of screening both inside [17] and outside a BCSP [7]. Other individual level characteristics include residential instability [18], being an immigrant [19], physical disability [20], and having one or more chronic diseases [21]. Only a relatively small amount of studies are dedicated to exploration of the determinants of screening outside a BCSP. Regular visits to a gynecologist, being employed and low esteem of the quality of the population screening program are associated with an increased attendance to screening outside a BCSP [6, 7, 22]. However, these studies only depend on self-reported data from health surveys or focus group discussions.

There is a paucity of studies that have investigated the determinants of screening coverage in a setting that has BC screening in and outside BCSP. The aim of this study therefore was to evaluate the factors associated with the coverage rate of mammography screening and factors that contribute to women’s choice of screening in and outside the BCSP using municipality level aggregated data.

## Methods

### Screening in Flanders

Flanders, the most populated region of Belgium, established a BCSP for women aged 50–69 in 2001 [5]. The organization and implementation of mammography screening in and outside the BCSP in Flanders have been described in detail elsewhere [5, 23, 24]. Briefly, in BCSP, every 2 years, eligible women aged 50–69 are actively recruited through a personalized invitation letter sent by the Center for Cancer Detection in Flanders with a fixed time and place for a digital mammography screening fully and directly paid by the health insurance system in Flanders. The Flemish program follows the European quality assurance guidelines [9]. Mammography screening outside the context of the BCSP can be accessed by a referral from a general practitioner (GP) or a gynecologist, is not fully covered by health insurance [5], and does not systematically include quality-control activities (e.g. double reading) [5]. Since 2016, women who received reimbursement for mammography in the last 2 years from the health insurance or have been diagnosed with BC in the last 10 years in the Flemish health care system are not invited for the population screening program.

### Data description

Municipality level screening coverage in 2008–2016 was calculated using data from the Center for Cancer Detection in Flanders [25]. Municipalities that have no missing values of the number of screened and non-screened women were included in the study. Independent variables at the municipality level of 2008–2016 were derived from the database of the Flemish provincial authorities and linked to data of the screening coverage. We included only the variables that were publicly available in order to reduce the bias that may be induced by the selection of variables [26].

### Privacy considerations

Privacy was warranted since only aggregated data were available at municipality level and for municipalities with less than 5 screened women overall or in one of the four age groups (50–54, 55–59, 60–64, and 65–69), a missing value was used.

### Main outcome

The main outcome of our analysis was the screening coverage rate inside and outside the BCSP from 2008 to 2016. The coverage rate was presented overall as a median value over all years and municipalities and stratified by age groups and the two screening strategies.

### Determinants considered

For an overview of the variables considered in the analysis, see Table 1. *Number of residents and population density* were defined as the total number of residents and the number of residents per km^2^ per municipality, respectively. *Natural balance* was defined as the natural growth per 1000 residents per municipality. *Residential stability* was indicated by the percentage of residents having the same address as the year before. *Non-Belgian nationality* was defined as the percentage of residents without a Belgian nationality per municipality. The socioeconomic status (SES) of residents was characterized by the following four proxy variables: (1) *Average household size* was defined as the average number of residents per households as a proxy for crowded housing conditions. (2)*Women with equivalent living wages* was defined as the percentage of women with equivalent living wages which is the minimum income awarded by the social welfare center. (3)*Share of borrowers with at least one overdue loan* was defined as the percentage of borrowers with at least one overdue loan per municipality where a high percentage was considered as a proxy for poverty; and (4)*Job seekers* were defined as the percentage of unemployed residents with waiting allowance or bridging allowance per municipality. Health status was indicated by residents aged 18–64 with *physical disability* or *status of diabetes*, and defined as the percentage of handicapped residents aged 18–64 and the percentage of residents with diabetes recognized by the health insurance system, respectively. Healthy behavior was indicated by *dental visit* defined as the percentage of residents having at least 2 visits at the dentist in 2 different years within a period of 3 calendar years per municipality.

**Table 1:** Social demographic parameters of Flanders per municipality in the period 2008–2016. In total 295 municipalities were included

	Median (P25-P75)
**Population and households**	
number of residents (10^5^ residents)	0.15 (0.10–0.22)
population density (1000 residents per km^2^)	0.41 (0.27–0.66)
natural balance (per 1000 residents)	0.86 (−0.76–2.43)
same address as last year (compared to all residents)%	92.50 (91.40–93.30)
non-Belgian nationality (compared to all residents)%	3.30 (2.10–6.00)
average household size	2.44 (2.37–2.51)
**Welfare and poverty (%)**	
women with equivalent living wages (compared to all women residents)	0.26 (0.18–0.41)
share of borrowers with at least one overdue loan (compared to all borrowers)	3.00 (2.50–3.80)
job seekers (compared to all residents)%	1.80 (1.40–2.20)
**Health and handicap (%)**	
person with physical disability18-64y (compared to all residents in 18-64y)	1.96 (1.57–2.58)
diabetes (compared to all residents)	5.10 (4.60–5.60)
dental visit (compared to all residents)	54.50 (51.30–57.70)

### Statistical analysis

Median value and interquartile range (IQR): p25-p75 were calculated for all continuous variables which were not normally distributed. The annual screening coverage rate inside and outside the BCSP was calculated as a median value over all years and municipalities and presented overall and stratified by four age groups: 50–54, 55–59, 60–64, and 65–69. To evaluate which determinants were related to the annual coverage of the two screening strategies, a logistic regression model with generalized estimating equations (GEE) was constructed to account for the correlation of repeated measurements of municipality level screening coverage rate and social demographic parameters. In the GEE model, the dependent variable was the municipality level coverage rate and the independent variables were the municipality level social demographic parameters as given in Table 1. A binary variable that indicated the type of screening strategy that the coverage rate referred to was provided and used as an independent variable. Odds ratios (OR) were reported with 95% confidence interval (CI). The effect of social demographic parameters was investigated by assessing a two-way interaction between the two screening strategies and the significant independent variables. All statistical analyses were performed using R version 3.6.0, and a two-sided *P* < 0.05 was considered statistically significant.

## Results

**Fig. 1:**
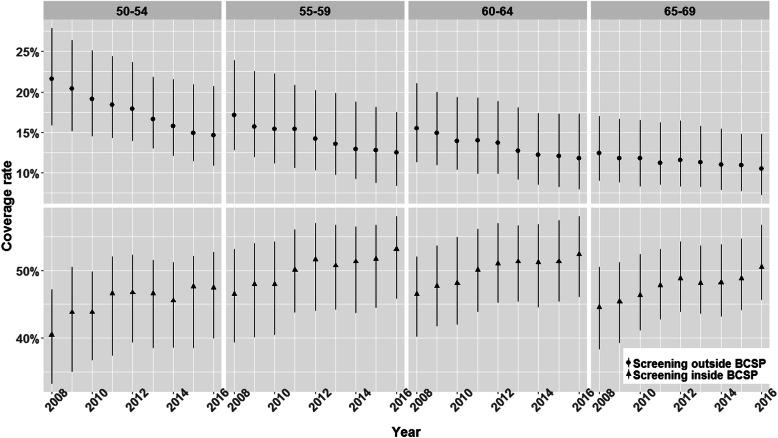
Median (P25-P75) screening coverage rate of 295 municipalities by age groups in 2008–2016

We included 295 of the 308 municipalities in Flanders that reported full data of the number of screened women of the two screening strategies in all age groups in 2008–2016. The median percentages of all included social demographic parameters over all years and municipalities are shown in Table 1.The overall median coverage of all years and municipalities of both screening strategies combined was 60.90%. The median coverage rates of all years and municipalities inside and outside the BCSP were 48.40% (IQR: 41.50–54.40%) and 14.10% (IQR: 9.80–19.80%) respectively, Table 2. The median coverage of screening outside the BCSP decreased from 2008 to 2016, especially in the youngest age group, while an increase of screening coverage inside the BCSP was seen in all age groups, Fig. 1.

**Table 2:** Median screening coverage (P25-P27) in Flanders

	Screening coverage (%): Median (P25-P75)	
	Population BC screening	Non-population BC screening
Overall	48.40 (41.50–54.40)	14.10 (9.80–19.80)
Age group		
50–54 year	45.40 (37.50–51.30)	17.50 (13.00–24.10)
55–59 year	50.10 (42.80–56.10)	14.30 (10.00–20.50)
60–64 year	50.10 (43.60–56.20)	13.50 (9.30–18.70)
65–69 year	47.80 (42.40–53.40)	11.40 (8.20–16.00)

From the univariate analysis it followed that significantly less women were screened outside the BCSP than inside the BCSP (OR: 0.184, 95% CI: 0.180–0.189). The probability of being screened in or outside the BCSP was positively associated with the average household size (OR: 1.282, 95% CI: 1.138–1.444), while negatively associated with the percentage of women with equivalent living wages (OR: 0.899, 95% CI: 0.855–0.945), the percentage of unemployed job seekers (OR: 0.961, 95% CI: 0.936–0.987) and population density (OR: 0.918, 95% CI: 0.888–0.949). (Table 3). After the adjustment for social demographic parameters in the multivariate analysis, the probability of being screened inside or outside the BCSP was only negatively associated with average household size (OR: 0.894, 95% CI: 0.809–0.988), population density (OR: 0.929, 95% CI: 0.906–0.952), and diabetes prevalence (OR: 0.964, 95% CI: 0.952–0.976) whereas positively associated with the percentage of unemployed job seekers (OR: 1.073, 95% CI: 1.051–1.095), and the percentage of residents with proper dental care (OR: 1.005, 95% CI: 1.003–1.007) (Table 4).

**Table 3:** Univariate analysis of the determinants of screening in or outside the population BC screening

Variable	Crude OR (95% CI)	***P*** value
**Year**	1.002 (0.996–1.008)	0.534
**Age group**		**< 0.001**
50–54 year	ref	
55–59 year	1.034 (0.993–1.077)	
60–64 year	1.014 (0.973–1.057)	
65–69 year	0.933 (0.894–0.973)	
**BC screening**		**< 0.001**
Population BC screening	ref	
Non-population BC screening	0.184 (0.180–0.189)	
**Population and households**		
number of residents (10^5^ residents)	0.962 (0.941–0.983)	**< 0.001**
population density (1000 residents per km^2^)	0.918 (0.888–0.949)	**< 0.001**
natural balance (per 1000 residents)	0.996 (0.990–1.002)	0.194
same address as last year (compared to all residents)	1.024 (1.013–1.035)	**< 0.001**
non-Belgian nationality (compared to all residents)	0.996 (0.993–0.999)	**0.016**
average household size	1.282 (1.138–1.444)	**< 0.001**
**Welfare and poverty**		
women with equivalent living wages (compared to all women)	0.899 (0.855–0.945)	**< 0.001**
share of borrowers with at least one overdue loan (compared to all borrowers)	0.970 (0.958–0.982)	**< 0.001**
job seekers (compared to all residents)	0.961 (0.936–0.987)	**0.004**
**Health and handicap**		
physical disability18-64y (compared to all residents of 18-64y)	1.003 (0.986–1.021)	0.701
diabetes (compared to all residents)	0.972 (0.954–0.991)	**0.003**
dental visit (compared to all residents)	1.009 (1.006–1.012)	**< 0.001**

**Table 4:** Multivariable analysis of the determinants of screening in or outside the population BC screening

Variable	Model 1^a^		Model 2^b^	
	Adjusted OR (95% CI)	***P*** value	Adjusted OR (95% CI)	***P*** value
**Age group**		**< 0.001**		**< 0.001**
50–54 year	ref		ref	
55–59 year	1.039 (1.015–1.065)		1.207 (1.179–1.235)	
60–64 year	1.018 (0.994–1.042)		1.217 (1.190–1.244)	
65–69 year	0.928 (0.907–0.949)		1.142 (1.117–1.167)	
**BC screening**		**< 0.001**		**< 0.001**
Population BC screening	ref		ref	
Non-population BC screening	0.224 (0.220–0.229)		0.303 (0.295–0.312)	
**Population and households**				
number of residents (10^5^ residents)	0.983 (0.965–1.001)	0.059	0.996 (0.985–1.007)	0.444
population density (1000 residents per km^2^)	0.929 (0.906–0.952)	**< 0.001**	0.647 (0.634–0.660)	**< 0.001**
same address as last year (compared to all residents)	1.008 (0.998–1.018)	0.123	1.005 (0.995–1.015)	0.309
non-Belgian nationality (compared to all residents)	1.0005 (0.9984–1.0025)	0.654	0.9997 (0.9982–1.0012)	0.719
average household size	0.894 (0.809–0.988)	**0.028**	0.580 (0.522–0.645)	**< 0.001**
**Welfare and poverty**				
women with equivalent living wages (compared to all women)	0.972 (0.934–1.012)	0.164	0.987 (0.947–1.029)	0.532
share of borrowers with at least one overdue loan (compared to all borrowers)	0.989 (0.976–1.002)	0.092	0.989 (0.978–1.001)	0.067
job seekers (compared to all residents)	1.073 (1.051–1.095)	**< 0.001**	1.250 (1.226–1.273)	**< 0.001**
**Health can handicap**				
diabetes (compared to all residents)	0.964 (0.952–0.976)	**< 0.001**	0.985 (0.973–0.997)	**0.016**
dental visit (compared to all residents)	1.005 (1.003–1.007)	**< 0.001**	1.016 (1.015–1.018)	**< 0.001**
**Interaction terms**				
age group × BC screening				**< 0.001**
NPS × 50–54 year			ref	
NPS × 55–59 year			0.668 (0.642–0.694)	
NPS × 60–64 year			0.612 (0.589–0.636)	
NPS × 65–69 year			0.554 (0.533–0.576)	
NPS × population density			2.528 (2.455–2.606)	**< 0.001**
NPS × average household size			3.797 (3.199–4.508)	**< 0.001**
NPS × job seekers			0.641 (0.624–0.658)	**< 0.001**
NPS × status of diabetes			0.942 (0.921–0.962)	**< 0.001**
NPS × dental visit			0.969 (0.967–0.972)	**< 0.001**

^a^model 1: multivariable regression model including all significant covariates of the univariate regression

^b^model 2: multivariable regression model including two-way interaction terms between screening strategies and the significant covariates in model 1

*NPS* Non-population BC screening

Contrary to the BCSP, the probability of being screened outside the BCSP was positively associated with being in a younger age group, a high population density (OR: 2.528, 95% CI: 2.455–2.606), and a larger households size (OR: 3.797, 95% CI: 3.199–4.508), and negatively associated with the diabetes prevalence (OR: 0.942, 95% CI: 0.921–0.962), the percentage of unemployed job seekers (OR: 0.641, 95% CI: 0.624–0.658) and the percentage of residents with proper dental care (OR: 0.969, 95% CI: 0.967–0.972) (Table 4).

## Discussion

In the present study, we assessed the coverage determinants of screening inside and outside the BCSP in Flanders. A median 48.4% of women aged 50–69 are screened by the BCSP which is significantly higher than the 14.1% of women screened outside the program. Working women in younger age group (50–54 years of age), and women living in crowded households with low dental care go less frequently to the screening, and if they go, they tend to be screened more frequently outside the context of the BCSP.

The total median coverage rate of 60.90% of screening inside and outside the BCSP is within the range of coverage levels of European countries (average: 48.2% (range: 19.4–88.9%)) [27]. The median coverage rate of the BCSP in Flanders of 48.4% is close to the coverage rate of the BCSP in countries such as France (52.8%) [6] and Switzerland (46.7%) [7, 27] and higher than in Serbia (38.0%) [28]. In these three countries there is screening in and outside the context of the BCSP. However, it is much lower than the coverage rate of the BCSP in some western and northern European countries like the United Kingdom (78.0%), the Netherlands (78.5%), and Norway (72.1%) [27] where only the BCSP is endorsed as the population screening strategy.

From 2006 to 2016, the coverage rate of BCSP increased while the coverage rate outside the BCSP decreased. This effect might be explained by public health campaigns via mass media and community education programs [24, 29], which increased the visibility and awareness of BCSP for the target population and their doctors [29, 30]. A decrease in screening coverage rate was observed from 17.50 to 11.40% for the individuals from age 50–54 to 65–69 years old in the screening outside the BCSP, whereas this pattern was not observed for the individuals in the BCSP. A similar pattern is also observed in countries like France [6, 31] and the United States of America [32] where both screening strategies are provided in large scale. A potential explanation can be that older women are more likely to attend the relatively fixed time and place of the BCSP than younger working women.

We found that living in crowded households, living in an area with high population density, and having a low dental care are associated with a lower probability of being screened. These three characteristics are all indicators for a low SES. People living in areas with a high population density tend to have a lower SES [33]. People living in crowded household are more likely to fall into income poverty [34]. As dental care is not fully covered by the health insurance system in Flanders [35], a low dental care indicates a lower SES [36]. Similar associations are also available in the literature regarding the increased BCSP coverage and increased dental care [19], less crowded household condition [14], and decreased population density [37].

Interestingly, women that are characterized by living in an area with high population density, living in a more crowded households, or having a low dental care tend to go more frequently for screening outside the BCSP. The reverse SES gradient in the use of screening in and outside BCSP was also seen in other settings where both screening strategies coexist [6, 7, 37]. An explanation for this phenomenon is that women with a higher SES are more likely to have a higher level of health literacy [38]. For these women, information regarding the importance of mammography screening and the systematic quality control is more likely to motivate them to participate in the BCSP [5] [29]. Another explanation is that poor employed women could have less flexible working time, which can conflict with the fixed working time of organized screening units [6, 7, 37, 39]. It has also been mentioned that areas with a higher population density have a lower population BC screening capacity (defined as the number of mammography facilities per 10,000 women) [40] and that in these areas there are more private clinics for opportunistic screening [37]. As a lower capacity of screening units can induce a longer waiting time and therefore a lower satisfaction of screening experience [5], low SES women living in these areas might be more likely to have negative screening experience and as a consequence prefer to go for screening outside the BCSP [22].

The strength of this study is that we examined determinants of coverage rate of screening in and outside the BCSP with longitudinal administrative data instead of self-reported screening uptake, which may induce recall bias. For that, regular collected and maintained administrative data of screening coverage outside the BCSP were applied. This enabled us to evaluate the determinants of the two coexisting screening strategies for BC and to better understand which further efforts are needed to improve the coverage of the BCSP in Flanders. However, our study had some limitations as well. First, a limitation of this study was the use of aggregated data, which reduced the options to evaluate correlation structures in the data [41]. Similarly, due to the aggregated data, a variation of coverage rate and the associated determinants within a municipality can be concealed. However, the association between the determinants and screening uptake in our study is consistent with other studies that applied neighborhood or individual level factors [13, 18, 19]. Second, proxy variables for SES were applied instead of income which can directly characterize SES of women. However, the proxy variables used are commonly applied and the magnitude and direction of the association between variables is consistent with the literature [6, 14, 18].

## Conclusion

A sizeable part of women attend screening outside the BCSP in Flanders. Women with low SES that are characterized by younger age, living in a high population density area, living in crowded households, or having low dental care, go less frequently to screening. If they go to screening, they are more likely to be screened outside the BCSP. Further efforts targeted on this group of women are needed to improve the coverage rate of the BCSP in Flanders.

## Data Availability

Breast cancer screening coverage dataset is available at https://bevolkingsonderzoek.incijfers.be/, variables regarding the determinants of screening coverage can be requested by contacting the Center for Cancer Detection in Flanders at www.bevolkingsonderzoek.be.

## Abbreviations

BC: Breast cancer
BCSP: Breast cancer screening program
GP: General practitioner
SES: Socioeconomic status
IQR: Interquartile range
GEE: Generalized estimating eqs.
OR: Odds ratios
CI: Confidence interval
